# Statistical Analysis of Tract-Tracing Experiments Demonstrates a Dense, Complex Cortical Network in the Mouse

**DOI:** 10.1371/journal.pcbi.1005104

**Published:** 2016-09-12

**Authors:** Rolf J. F. Ypma, Edward T. Bullmore

**Affiliations:** 1 Brain Mapping Unit, Department of Psychiatry, University of Cambridge, Cambridge, United Kingdom; 2 Hughes Hall, Cambridge, United Kingdom; 3 Behavioural and Clinical Neuroscience Institute, Department of Psychology, University of Cambridge, Cambridge, United Kingdom; 4 Cambridgeshire & Peterborough NHS Foundation Trust, Cambridge, United Kingdom; 5 ImmunoPsychiatry, Immuno-Inflammation Therapeutic Area Unit, GlaxoSmithKline, Stevenage, United Kingdom; Oxford University, UNITED KINGDOM

## Abstract

Anatomical tract tracing methods are the gold standard for estimating the weight of axonal connectivity between a pair of pre-defined brain regions. Large studies, comprising hundreds of experiments, have become feasible by automated methods. However, this comes at the cost of positive-mean noise making it difficult to detect weak connections, which are of particular interest as recent high resolution tract-tracing studies of the macaque have identified many more weak connections, adding up to greater connection density of cortical networks, than previously recognized. We propose a statistical framework that estimates connectivity weights and credibility intervals from multiple tract-tracing experiments. We model the observed signal as a log-normal distribution generated by a combination of tracer fluorescence and positive-mean noise, also accounting for injections into multiple regions. Using anterograde viral tract-tracing data provided by the Allen Institute for Brain Sciences, we estimate the connection density of the mouse intra-hemispheric cortical network to be 73% (95% credibility interval (CI): 71%, 75%); higher than previous estimates (40%). Inter-hemispheric density was estimated to be 59% (95% CI: 54%, 62%). The weakest estimable connections (about 6 orders of magnitude weaker than the strongest connections) are likely to represent only one or a few axons. These extremely weak connections are topologically more random and longer distance than the strongest connections, which are topologically more clustered and shorter distance (spatially clustered). Weak links do not substantially contribute to the global topology of a weighted brain graph, but incrementally increased topological integration of a binary graph. The topology of weak anatomical connections in the mouse brain, rigorously estimable down to the biological limit of a single axon between cortical areas in these data, suggests that they might confer functional advantages for integrative information processing and/or they might represent a stochastic factor in the development of the mouse connectome.

## Author Summary

Tract-tracing depends on active axonal transport of tracers between nerve cells, indicating the anatomical connectivity between areas of the brain. Recent advances in tract-tracing technology have enabled reconstruction of the connectome or wiring diagram of mammalian cerebral cortex. Here, we propose a novel statistical model to account for the noise arising from automation of tract-tracing measurements and from injections of tracer into multiple cortical areas simultaneously. On this basis, we find that the strength of anatomical connectivity in the mouse brain varies over six orders of magnitude, with the weakest links between regions approximately representing a few axons. Including all weak links above the statistical noise thresholds, we find that the connection density of the mouse connectome (73%) is greater than previously reported. Many of the complex topological and spatial properties of the mouse brain network emerge on the basis of the strongest axonal projections, whereas the weakest links have a more random organization. We conclude that inter-areal connections mediated by a few axons can be rigorously distinguished from experimental sources of noise in contemporary tract tracing data. Such weak links could support integrated functions of the mouse brain network and/or could represent an element of randomness in its formation.

## Introduction

Recently, there has been much interest in the connectome perspective of the brain, which aims to map the entire set of connections or interactions between brain regions, rather than the more traditional focus on individual regions and their connectivity [1, 2]. This view may offer new insights both into general rules underlying the pattern of connections of the healthy developing brain, and how these patterns are disturbed in disorders and disease [3–5]. Non-invasive techniques such as functional or diffusion magnetic resonance imaging, electroencephalography and magnetoencephalography allow for measuring these networks at the whole-brain scale. However, these techniques only indirectly measure the actual axonal connectivity between brain regions. Despite major advances in sophisticated statistical and computational methods to process these data, direct interpretation in terms of neurons and axons is infeasible. For this reason, there is great interest in carrying out such analyses in animal model systems where more direct measurements can be made.

In mammals, tract tracing is considered the gold standard for assessing axonal connectivity. This invasive technique allows for quantitative measurement of the strength or weight of axonal projections between cortical areas. However, the technique has been challenging to scale up, and historically most tract-tracing studies have necessarily focused on connectivity of a few regions experimentally studied. Meta-analytic collation of multiple primary studies in the literature was used for pioneering graph theoretical analysis of the cat and the macaque connectomes [6]; but there are many technical complications in combining published tract-tracing data, such as inconsistent usage of atlases and methodology across primary studies [7]. Recent years, however, have seen several institutes publish large-scale efforts to comprehensively map axonal connectivity in the mouse [8, 9] and the macaque [10] by collecting high resolution images of tracer propagation from a large number of injection experiments coordinated by a standard protocol. Initial analyses have already shown the potential value of these “next generation” tract-tracing datasets for informing our understanding of the organization of the mammalian connectome [11–15] and for identifying a large number of so-called “new found projections” or axonal projections below the limit of resolution of historical methods.

The gold standard for assessing connectivity from microscopy images is expert visual inspection and manual demarcation of signal from background noise. This process of expert curation of the images has been used to define axonal connections in a large dataset of tract-tracing experiments in the macaque [10, 16]. But expert curation is time-consuming and observer dependent so the Allen Institute for Brain Sciences (AIBS) has taken a more computational approach in analysing their large tract-tracing dataset in the mouse, using automated image analysis algorithms to quantify the amount of signal present in each target region [9]. This algorithmic approach may suffer from decreased sensitivity, i.e. greater risk of failing to detect a true signal when it is present, or decreased specificity, i.e. a greater risk of failing to rule out a true signal when it is absent.

Statistically principled methods are currently lacking to estimate connectivity from these datasets. However, the published large datasets contain repeated measurements for many connections. These allow for estimation of variability, an indicator of experimental noise and inter-animal differences, and can thus be used for principled estimation of uncertainty in connectivity estimates. In particular, the mouse tract-tracing data published by the AIBS might benefit from a more formal statistical approach, since the segmentation algorithm used for automated analysis was found to have low specificity or high false positive rate [9], when algorithmic assessment was benchmarked against expert evaluation of a subset of experiments. Moreover, such a method may be used to assess the impact of incomplete data, as even the comprehensive AIBS dataset does not include primary injections into all cortical regions.

An open question in systems neuroscience is what percentage of brain regions that could be connected are, in fact, connected. This is fundamentally a question about specificity and sensitivity of detection of connections or links. Recently, it has been claimed that 62% of all possible interregional intrahemispheric connections in macaque cortex exist [10, 17] (66% for a fully investigated subset of regions), which is much higher than previous estimates of 15%-45% cortical connection density [18–20], perhaps reflecting the superior sensitivity of next-generation tract-tracing technologies. Biologically, one would expect network density to increase with decreasing brain size, both because in smaller brains long-distance connections are relatively less costly [21], and because smaller brains generally have fewer regions. However, recent estimates of cortical network density are low for mouse [34%-41% (isocortex) [8]; 35.4%-53.5% (Table S5 from [9])] and rat [45.1% [13]].

Here, we provide a statistical framework for estimating axonal connectivity in large-scale tract-tracing datasets. First, we explore the reliability of different tract-tracing datasets by assessing the variability of repeated measurements of axonal connectivity in (i) the collated Cocomac macaque dataset (collated macaque) [22]; (ii) the multi-experiment dataset by Markov et al. on macaque (multi-experiment macaque) [10]: and (iii) the multi-experiment dataset by the AIBS on the mouse (multi-experiment mouse) [9]. Second, we propose a statistical model to estimate the mouse brain network from algorithmically segmented data with positive-mean noise and co-injected regions, estimating both connectivity weights and associated credibility intervals. Third, we use this approach to rigorously estimate cortical network density of the mouse brain and to enable graph theoretical analysis of connectome topology over the full range of axonal connection weights.

## Materials and Methods

### Data

#### Multi-experiment mouse dataset

**Fig 1:**
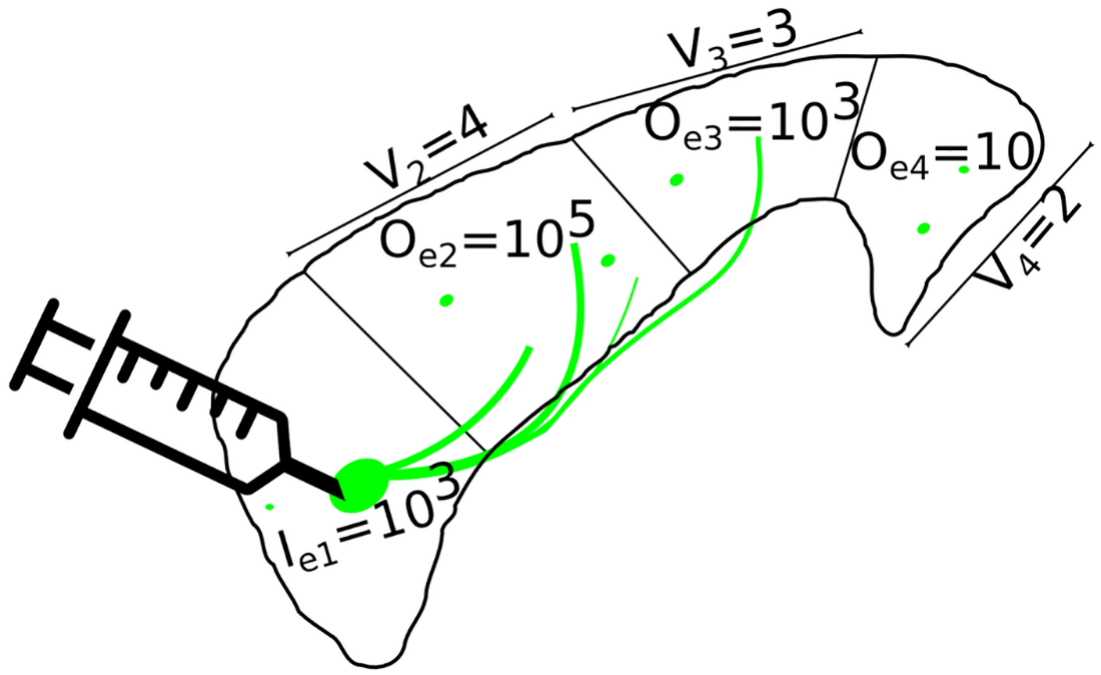
Schematic illustration of an experiment *e*. Tracer is injected into a volume *I*_*ei*_ of the source region *i*. Signal *O*_*ej*_ is then algorithmically segmented for each target region *j*. Signal density *O*_*ej*_/*V*_*j*_ can be found by dividing by target region volume *V*_*j*_. The connection weight is found by further normalizing by the total injection volume: *O*_*ej*_/*V*_*j*_/∑_*i*_ *I*_*ei*_, a metric known as normalized connection density [9]. Expert curation of algorithmically segmented data identified false positives for 20 experiments, such as illustrated for region 4.

The Allen Institute for Brain Sciences (connectivity.brain-map.org) provides data on 489 injections of an anterograde adenoviral tracer into the right hemisphere of wild type mice; 127 of these injections were into the cerebral cortex. These data were registered to a 50 *μ*m resolution atlas, based on the average brain of 1675 specimens, and then parcellated into 43 cortical regions (86 in total), each of which subtended the full thickness of cortex, in each cerebral hemisphere (S1 Table). Expert visual evaluation of the data from each experimental injection *e*, and each source region *i*, measured the total injection volume *I*_*ei*_ as the volume containing fluorescently stained cell bodies. The total tracer signal *O*_*ej*_ was measured in each target region *j* by an automatic segmentation algorithm, which estimates the total volume of the region showing fluorescent signal. Fig 1 illustrates these quantities. Thus, the experimental data consist of a {127 × 86} input matrix of *I* values, and a {127 × 86} output matrix of *O* values. As injections are generally contained in a small number of regions, only 4% of the *I* values were greater than zero. However, some level of signal was algorithmically detected in most target regions following most injections, so 98% of the *O* values were greater than zero. This high proportion of non-zero elements in the *O* matrix is consistent with a high density of “true” anatomical connections between regions and/or a high false positive rate of the algorithm.

Twenty of the 461 experiments were chosen randomly by the AIBS to be fully analyzed by expert human assessment of the data. These experiments roughly matched the distribution of injection volumes over the entire dataset. Four annotators assessed five experiments each, in order to provide a gold standard for analysis of the false positive rate of the algorithm for automated assessment of the majority of the experiments. These data have previously been presented in processed form in Figure S7 in [9]. They were kindly provided to us by the AIBS, and we make them available here (with permission) as S2 Table. For each of the 86 target regions, both the segmentation algorithm and a human expert evaluated whether the region contained axonal tracer. For these 20 × 86 = 1720 evaluations, the algorithm detected signal 1616 (94%) times. However, 773 (48%) of these algorithmically positive evaluations were not endorsed by expert human assessment and were therefore defined as false positives, as can be calculated from S2 Table. No false negatives were found.

#### Multi-experiment macaque dataset

Markov et al. (2012) performed a total of 39 injections of retrograde tracers (fast blue and diamidino yellow) into 29 regions of the cerebral cortex of the macaque monkey, using a 91-region atlas defined by histological criteria and atlas-based landmarks. All injections were fully contained within a single source region. Following each injection, they semi-manually counted the number of stained neurons in each of the 91 target regions of one hemisphere (core-nets.org). 57% of these {39 × 91} measurements were greater than zero.

#### Meta-analytically collated macaque dataset

The CoCoMac dataset (cocomac.g-node.org) on **co**rtical **co**nnectivity of the **mac**aque monkey comprises primary literature reports on 725 tracer injections into various brain regions [19], algorithmically registered post hoc to the FV91 atlas [19, 23]. In contrast to the other two datasets, it provides measurements on specific region pairs, rather than measurements on all target regions for each injection. The connection between each region pair is typically classified as absent or present, and in some cases additionally assigned a strength value on an ordinal scale (1, 2 or 3). In total, 11452 measurements are provided, of which 3079 (27%) are weighted.

### Definitions of axonal connectivity weight

The connectivity weight can be defined in different ways. All definitions depend on the total volume of signal *O*_*ej*_ found in the target region, but differ in how this value is normalized [9, 12]. Preferably, our definition of weight would not depend on the total volume *V*_*i*_, *V*_*j*_ of regions involved. We will therefore estimate the “normalized connection density” *n*_*ij*_: the amount of signal expected per unit volume of target region *j*, after injection of one unit volume of source region *i*. *n*_*ij*_ can be estimated from an experiment as $\frac{O_{ej}}{V_{j}I_{ei}}$. However, data on the amount of tracer injected, *I*_*ei*_, are only available for the multi-experiment mouse dataset. Therefore, in analyses comparing the different datasets, we will use the fractional weight *f*_*ij*_, the fraction of the total signal measured in target regions that is contained in region *j* after injection of region *i*, introduced as the “fraction of labeled neurons” by Markov et al [16]. *f*_*ij*_ can be estimated from an experiment as *O*_*ej*_/∑_*k* ≠ *i*_ *O*_*ek*_.

### Variability of axonal connectivity weights

For each of these three datasets, we explored the variability of the measured weight of connectivity between regions. We focus on the continuous variable of weight rather than simply the (binary) presence or absence of connections as the mouse dataset shows an extremely low variability of binary connections (i.e. *O*_*ej*_ > 0), due to its high false positive rate. For the mouse data, and the multi-experiment macaque data, we log-transformed the weights, to make them more comparable to the ordinal weights {1, 2, 3} assigned to each connection in the collated macaque dataset.

We constructed a measure of variability *V* as follows. First, we selected all pairs of regions for which at least one non-zero value was measured, and computed the mean connection weight *m*_*ij*_ for each pair. Second, we selected all pairs of regions for which at least two non-zero values were measured, and for each pair calculated the variance *v*_*ij*_ of the measured values. Last, we defined our measure of variability as the average of these variances, divided by the variance of the means

$$\begin{array}{c} V=\frac{E(\bar{v})}{\text{Var}(\bar{m})} \end{array}$$ (1)

This measure represents the variance in repeated measurements of the weight of the same connection scaled by the overall variance measured across all weights in the dataset. Note that this measure is invariant to affine transformations of the connection weights, which is important as the collated macaque labels {1, 2, 3} could be arbitrarily scaled to fit with more continuously quantitative measures of connection weight. To examine whether weaker connections are more variable, we compute *V*_0.5_ and *V*_0.1_, defined as above but with the variance taken over the 50% and 10% weakest weights. Finally, we perform sensitivity analyses on this metric to test robustness of results to selection of region pairs and exclusion of zero weights (S1 Appendix).

#### Multi-experiment mouse dataset

We selected all regions that have at least one injection for which more than 50% of the injection volume is into that region, i.e., *I*_*ei*_ > 0.5∑_*k*_ *I*_*ek*_. For each of these injections, we compute the log-transformed total fractional weight *f*_*ij*_: $\log_{10}\frac{O_{ej}}{\sum_{k\neq i}O_{ek}}$ in each of the 85 target regions *j*, thus excluding the injected region.

#### Multi-experiment macaque dataset

Markov et al. (2012) injected four regions (V1, V2, V4 and 10) at least twice, specifically to assess variability. For each of these 14 experiments, we log_10_ transform the fraction of neurons *f*_*ij*_ labeled in each region.

#### Meta-analytically collated macaque dataset

To calculate the variance in the mean connection weights, we considered only those regional pairs for which at least one non-zero connection weight {1, 2, 3} was available. To calculate the mean of the variance in the connection weights, we considered only those regional pairs for which at least two non-zero connection weights {1, 2, 3} were available.

### Estimation of mouse brain connectome

#### Model in the absence of noise

We modelled the log-transformed normalized connection density $\log_{10}\left(\frac{O_{ej}}{V_{j}I_{ei}}\right)$ [9] in target region *j* due to signal propagation from an injection of 1 mm^3^ of tracer in the source region *i* as log-normally distributed. This distribution is characterised by two parameters. The mean *μ*_*ij*_ (on a log scale) represents the edge weight for each connection on average over animals. The standard deviation *σ*, representing inter-animal variability, was assumed to be equal for all connections. If the injection is contained entirely in a single region, and in the absence of noise, we thus have:

$$\begin{array}{c} \frac{O_{ej}}{V_{j}I_{ei}}∼LN\left(\mu_{ij},\sigma \right) \end{array}$$ (2)

where *LN* is the lognormal distribution. As *σ* is informed by regions that were injected multiple times, we would expect its estimate to be close to the standard deviation of the normalized densities when we take all experiments *e* that are into the same source region. Thus, we performed a quick check of our estimate of *σ*, by computing for each target region *j* the standard deviation of $\frac{O_{ej}}{V_{j}I_{ei}}$ for each of the three most often injected regions *i*, where we again took experiments *e* that had at least 50% of injection volume in region *i*. For each of the three injected regions *i*, we then took the median over these values, and compare these to *σ*.

#### False positives due to automated segmentation

Automatic segmentation of fluorescent signal is a daunting problem to solve [24]. Even the sophisticated segmentation algorithm employed in the AIBS dataset has relatively low selectivity, i.e. it often identifies a tracer signal in regions where none is recognisable by expert curation. However, these noise-generated values are usually small and can therefore be considered as a noise threshold below which one cannot distinguish between an absent connection and a very weak connection. Previous methods have accounted for false positives by using a simple cut-off value, setting all values below this cut-off equal to 0 [9, 12]. We improved on this by using a probabilistic, region-specific threshold, constructed by quantifying the distribution of weights in the absence of a connection. We modelled the probability density function *g*_*j*_ of noise-generated values for region *j* (and its contralateral homologue) by non-parametric kernel-based estimation based on the axonal connectivity weights classified as false positive by expert curation of 20 experiments. To show this is a sensible approach, we checked three expectations.

Firstly, we expected that false positive signal values should be independent of the injection volume, and correspond to the density rather than the total volume of signal. We tested this by asking which of three possible metrics of connectivity best separated true positive from false positive connections: signal volume *O*_*ej*_; signal density $\frac{O_{ej}}{V_{j}}$; or normalized connection density $\frac{O_{ej}}{V_{j}\sum_{i}I_{ei}}$. For each region, we computed these three measures for the 20 connections measured in the 20 experiments, and computed the normalized test statistic *U* from the Mann-Whitney *U* test. This statistic compares, for a given measure, all possible pairs of a true and a false positive value, and represents the fraction of times the measure is larger for the true positive. The ideal connectivity metric would yield *U* = 1 by this analysis. Secondly, we expected these values to be different between regions, as some tissue may be more prone to errors than other. We used the Kruskal-Wallis test to test the null hypothesis that false positive signal densities were similar between regions. Thirdly, we expected contralateral homologous cortical regions to show a similar distribution of noise-generated values. We tested this using pair-wise Mann-Whitney tests. As the individual tests have low statistical power, we compared the number of homologous pairs found to be significantly different (*P* < 0.1) to the number expected for random pairs, obtained by 1000 random permutations of the regional labels of one hemisphere.

#### Co-injection of tracer into more than one source region

In the AIBS dataset, tracer injections are often not fully contained in one region of mouse cortex, with some spillover of tracer into adjacent cortical areas. This means the signal subsequently propagates throughout the brain via the connections of both injected regions. We could model the propagated signal as a convolution of the contributions of the two injected regions. However, this assumes independence between the connections of the co-injected regions. As the co-injected regions will be spatially adjacent, and in the same animal, an assumption of independence seemed implausible. We therefore modeled the signal arising from co-injection as being generated from a region that has connectivity properties intermediate between the two injected regions:

$$\begin{array}{c} \mu_{ej}=\log_{10}\left(\sum_{i}[\frac{I_{ei}}{\sum_{k}I_{ek}}10^{\mu_{ij}}]\right). \end{array}$$ (3)

where both sums are over all regions. This model ensures that the expected value of the log-transformed density generated by the combination of regions is equal to the sum of the expected values for the regions separately.

#### Zero values

In the mouse dataset, two percent of all measured values *O*_*ej*_ were equal to zero. We assumed that zero values were either truly 0 or too weak to be detected by the tract-tracing technology and automated segmentation algorithm. We approximated the lowest density measurable as the lowest non-zero density measured:

$$\begin{array}{c} l=\min_{e}\min_{\left\{j:O_{ej}>0\right\}}\frac{O_{ej}}{V_{j}} \end{array}$$ (4)

Thus, we assumed that an observed density of zero, $\frac{O_{ej}}{V_{j}}=0$, should be interpreted as observing a density of at most the smallest density: $\frac{O_{ej}}{V_{j}}<l$.

#### Likelihood

Each measurement arises from the combination of incorrectly segmented signal (noise) and correctly identified signal indicating an axonal projection from the source to the target region. As we are working with log transformed data, we approximated the resulting convolution by assuming that the contribution of one of these factors dominates. Since we cannot differentiate fluorescent signal in a region directly due to an injection from that due to signal propagation, we cannot evaluate the connection to any target region that is itself a source region. Therefore, we did not consider any target region *j* that was itself co-injected, i.e. *I*_*ej*_ > 0. Thus, the full likelihood becomes

$$\begin{array}{ccc} L(\bar{O},\bar{I}|\bar{\mu },\sigma ) & = & \Pi_{e}\Pi_{\left\{j:I_{ej}=0\right\}}1_{O_{ej}>0}[G_{j}\left(\frac{O_{ej}}{V_{j}}\right)f\left(\frac{O_{ej}}{V_{j}I_{e}},\mu_{ej},\sigma \right)+g_{j}\left(\frac{O_{ej}}{V_{j}}\right)F\left(\frac{O_{ej}}{V_{j}I_{e}},\mu_{ej},\sigma \right)]\\ & & +1_{O_{ej}=0}F\left(\frac{l}{I_{e}},\mu_{ej},\sigma \right)G_{j}\left(l\right) \end{array}$$ (5)

where 1 is the indicator function, *f* and *F* are the probability and cumulative density functions of the log-normal distribution, *g*_*j*_ and *G*_*j*_ are the probability and cumulative density functions of the noise distribution for region *j*, and *I*_*e*_ = ∑_*i*_ *I*_*ei*_ is the total volume of injection for experiment *e*. Note that by the nature of the data, no distinction can be made between a connection that would generate less signal than the noise-generated values, and the absence of a connection.

#### Computation

The contributions of the different experiments are not always independent (because some experiments have co-injected more than one region), so we used a Monte Carlo Markov Chain (MCMC) to estimate the posterior distributions of $\bar{\mu }$ and *σ*, assuming uniform priors, [-20, 10], (0, 1000] (see S1 Appendix for a sensitivity analysis using informative priors). We obtained point estimates for $\bar{\mu }$ and *σ* by taking the median of the relevant marginal distributions. This estimator minimizes the absolute error and is robust to a long tail in the distribution. We ran the chain for 10^6^ iterations, proposing a new value for each parameter in each iteration, and sampled at every 200 iterations after an initial burn-in of 10^5^ iterations. We visually checked convergence of parameters. We ran 5 independent chains and checked these resulted in comparable distributions. Finally, our parameter estimation used the union of the samples from these 5 chains. Code for all analyses is available from https://github.com/rjfy2/tracer_statistics.

### Thresholds

Two probability thresholds exist in the multi-experiment mouse tract-tracing data, which put a lower bound on the weight at which connections can still be identified. These are the noise threshold and the co-injection threshold. The noise threshold is the result of incorrectly identified signal in the absence of a connection. We quantify its region-specific probability density function *g*_*j*_ as above. This distribution describes the density values to be expected for region *j* in the absence of a connection. A connection can only be identified if an injection into the source region generates a density in the target region that is higher than this noise threshold. The density generated depends both on the connection weight *μ*_*ij*_ and the injection volume *I*_*ei*_, as an increase in either will increase the expected density due to this connection: $\log_{10}\left(I_{ei}10^{\mu_{ij}}\right)$. We thus compute the noise threshold $T_{n}^{x}\left(i,j\right)$ as the minimum connection strength *μ*_*ij*_ needed such that the expected density of axonal connectivity of region *j* for at least one experiment is larger than the *x*th percentile of the noise distribution *g*:

$$\begin{array}{c} T_{n}^{x}\left(i,j\right)=\log_{10}\left(\min_{e}\frac{10^{g_{j}^{x}}}{I_{ei}}\right) \end{array}$$

with $g_{j}^{x}$ the *x*th percentile of *g*_*j*_.

The co-injection threshold is due to injections not limited to one region. Such co-injections lead to a threshold very similar to the noise threshold described above. For example, if both region *i*_1_ and *i*_2_ are injected, a strong connection of *i*_1_ to region *j* would mask the connection from *i*_2_ to *j*. To make this effect visible, we calculate the co-injection threshold *T*_*c*_(*i*, *j*) as the minimum *μ*_*ij*_ needed to ensure that, for at least one experiment, the expected signal due to this connection is larger than the expected signal due to all other connections:

$$\begin{array}{c} T_{c}\left(i,j\right)=\log_{10}\left(\min_{e}\frac{\sum_{k\neq i}I_{ek}10^{\mu_{kj}}}{I_{ei}}\right) \end{array}$$ (6)

We obtain a distribution of *T*_*c*_(*i*, *j*) by computing this statistic for each sample from the MCMC, and obtain the 90% credibility interval (CI) by taking the appropriate percentiles.

### Cumulative network density estimation

We estimate the network density function *d*(*x*), defined as the percentage of all possible connections that are of at least weight *x*. We estimate this function separately for intra-hemispheric and for inter-hemispheric connections. To facilitate comparison of the datasets, we here take the weight to be the fractional weight *f*_*ij*_ introduced before, which can be obtained from our estimated parameters as $\frac{V_{i}10^{\mu_{ij}}}{\sum_{k}V_{k}10^{\mu_{kj}}}$. *d*(*x*) gives the network density of the brain network if a cut-off of *x* is employed, i.e. setting all edges with weight smaller than *x* to 0. *d* is a monotonously decreasing function, and lim_*x* → ∞_ *d*(−*x*) is the total network density. As there is no information about the weight of a connection if it is weaker than its associated thresholds, we compute *d*(*x*) using only those connections *C*(*x*) for which both the median of its noise threshold and of its co-injection threshold are smaller than *x*:

$$\begin{array}{c} d\left(x\right)=\frac{1}{\left|C(x)\right|}\sum_{\left(i,j)\in C(x\right)}1_{f_{ij}>x} \end{array}$$ (7)

with 1 the indicator function, and |*C*(*x*)| the size of the set *C*(*x*). *d*(*x*) can be calculated for each sample of the posterior from the MCMC run, thus providing the uncertainty in the network density estimates. To obtain a point estimate and CI for the overall network density, we evaluate *d*(*x*) at the *x* such that exactly 50% of connections have a threshold higher than *x*, i.e. at the point where still half of connections can be measured. This choice balances the competing requirements of obtaining the network density at the finest level, i.e. minimal *x*, and basing the network density estimate on as many connections as possible, i.e. maximal *x*.

The multi-experiment macaque dataset was expertly curated and, by definition, contains no demonstrable false positives. Furthermore, all injections are into a single region, so there is no co-injection threshold to consider. We thus estimate the *f*_*ij*_ as above, assuming no noise, obtaining the credibility interval for each connection weight. As only a subset of projecting neurons are counted, there is a threshold to consider; the smallest weight measurable for each experiment is 1/(number of neurons measured). Therefore, for each level *x*, we compute *d*(*x*) as before from all regions *i* such that the maximum number of neurons measured for any injection into this region is at least 1/*x*.

Finally, we should note that not all regions were (sufficiently) injected to estimate connectivity, both for mouse (31/43 regions injected) and macaque (29/91 regions injected). This incompleteness may cause a bias, e.g. regions are both more likely to be injected and to have many connections when they are larger. In S1 Appendix we assess the possible bias caused by the preferential injection of larger, more connected regions, by estimating the connectivity of the uninjected regions using regression on simple anatomical properties.

### Biological interpretation

#### Multi-experiment mouse dataset

The measurements constitute the volume of tissue estimated to show fluorescent signal, which corresponds to the volume of the target region occupied by axons projecting from the injected (source) region. This means that (i) the signal is not equivalent to synaptic connectivity as we cannot directly observe the terminal arbors; and (ii) the signal does not directly correspond to the number of projecting neurons, since the thickness and length of projecting axons can differ substantially between target regions. However, tracer signal is regarded as a reliable and valid proxy of both synaptic connectivity and the number of axonal projections. We can further increase interpretability by noting that the estimated number of neurons in the mouse hemisphere is 4 × 10^6^ [25]. Only a fraction *r* ≤ 1 of these neurons project to another region. Thus, when all neurons in the source region are injected, the average number of neurons projecting from the source region is $\frac{4\times 10^{6}}{43}r$, and the average percentage of signal contributed by a single axonal projection is $\frac{43}{r4\times 10^{6}}$. While we do not know *r*, we can take $\frac{43}{4\times 10^{6}}$ as a lower bound on this average percentage.

#### Multi-experiment macaque dataset

The numbers of retrogradely labelled neuronal cell bodies enumerated by expert curation are likely an underestimate of the total number of neurons projecting to the injected region. This is because (i) retrograde tracer may not have propagated back to all projecting neurons, (ii) not all brain slices were curated [10], and (iii) injections do not cover the whole injected region. The macaque cortex is estimated to contain 1.5 × 10^9^ neurons per hemisphere ([26]: 1.3 × 10^9^, [27]: 1.7 × 10^9^). Thus, the average percentage of signal contributed by one neuron is $\frac{91}{r1.5\times 10^{9}}$, where *r* is again the unknown fraction of neurons projecting to another cortical region.

### Topological analysis of weak connections

To better understand the possible role of the weak but above-threshold connections, we perform two graph theoretical analyses on the estimated mouse connectome. Graph theory is a mathematical discipline that abstractly represents and analyses the mouse brain network as a set of nodes (cortical regions) and edges (axonal connections) between them.

First, we separately investigated the set of 5% weakest and 5% strongest above-threshold connections. We mapped these connections in anatomical space and compared their spatial distance distribution, using the Euclidean distance between regional centroids.

Second, we estimated the topological metric of global efficiency for the whole network, using both a weighted graph that considers the estimated connection weights, and a binary graph model network where connectivity weights are thresholded so that edges are either absent or present. We investigated how global efficiency changes as edges are deleted below a continuously variable threshold weight. This procedure is very similar to the calculation of the network density *d*(*x*) above, except that the metric requires that all connection weights are known. We therefore restrict our analysis to those target regions that are also the source region for at least one experiment. We make the additional assumption that connections are identical for contralateral homologue regions, i.e. if *i* and *k* are homologues, and so are *j* and *l*, we have *μ*_*ij*_ = *μ*_*kl*_. As 31 regions were injected with at least half of total injection volume in the mouse cortex, we retain 62 regions and 62 × 61 connections.

For arbitrary threshold *x*, we can then construct the network whose edge weights *e*_*ij*_ are given by

$$\begin{array}{c} e_{ij}\left(x\right)=\{\begin{array}{cc} 10^{\mu_{ij}}, & \text{if}\;\mu_{ij}>x\\ 0, & \text{otherwise} \end{array} \end{array}$$ (8)

We define the distance between two nodes as the inverse, i.e. $\frac{1}{e_{ij}}$, which is infinite when the weight is zero. A shortest path between any two nodes *A*, *B* is then a sequence of nodes *n*_1_, …, *n*_*k*_ such that *n*_1_ = *A*, *n*_*k*_ = *B*, *e*_*n*_*i*_*n*_*i*+1__ > 0, *i* = 1, …, *k* − 1 and the length of the path $L_{AB}=\sum_{i=1}^{k-1}\frac{1}{e_{n_{i}n_{i+1}}}$ is minimal. Global efficiency is defined as the average inverse shortest path length [28]

$$\begin{array}{c} G=\frac{1}{N(N-1)}\sum_{A\neq B}\frac{1}{L_{AB}} \end{array}$$ (9)

where *N* is the number of nodes in the network.

We also compute the fractional size of the largest component as a function of *x*. A component of the network is a subnetwork in which all nodes are directly or indirectly connected by edges. A fully connected network has only one component; in a network without edges each node is a component. The size of the largest connected component is the number of nodes it contains, which is a number between 1 and the total number of nodes in the network; divided by the number of nodes, this is the fractional size of the largest component, which ranges from $\frac{1}{N}$ to 1.

## Results

### Variability of axonal connectivity weights

**Fig 2:**
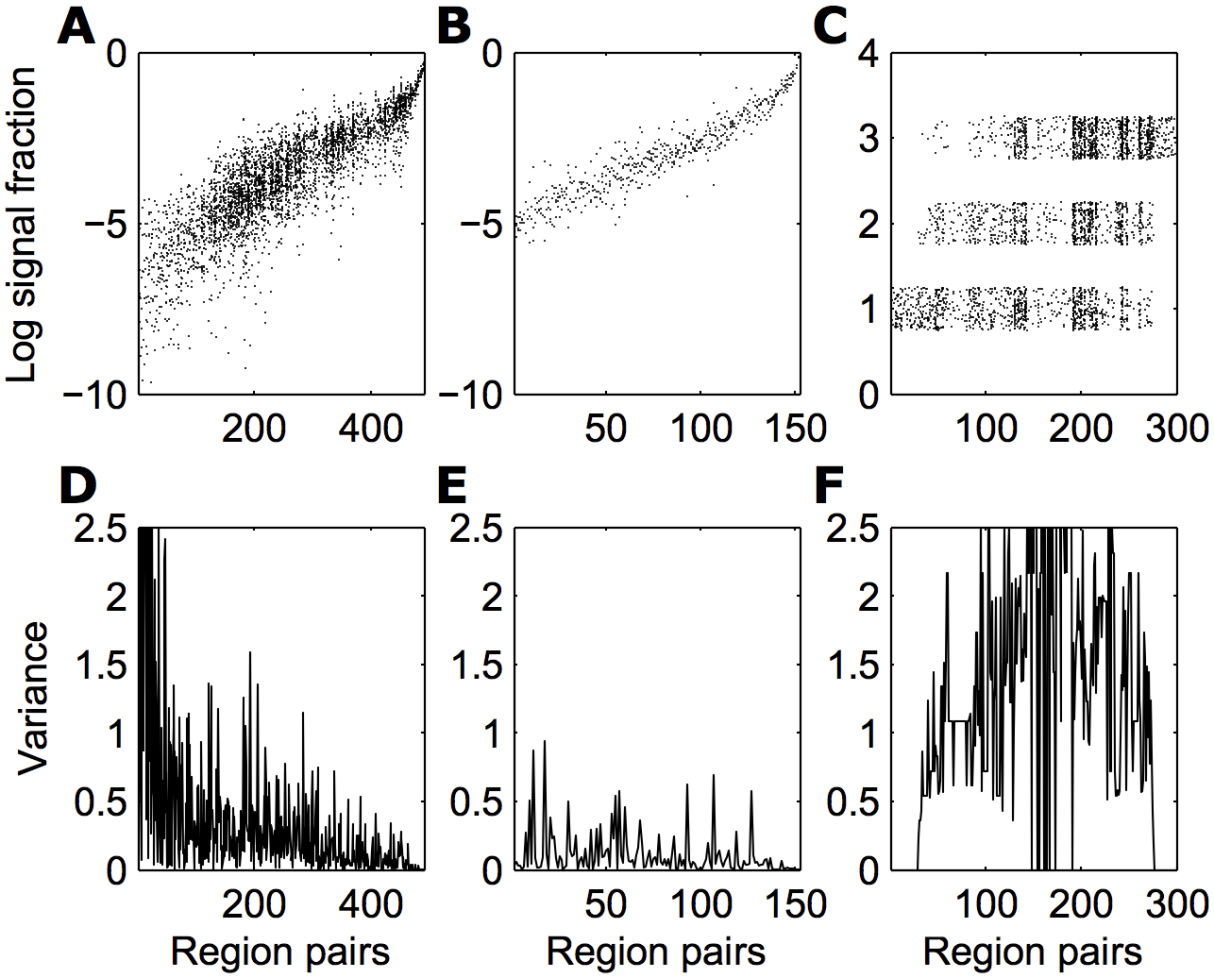
Variability of connection weights. Fraction of signal is shown for all pairs of regions with at least two non-zero values, for (A) the AIBS mouse dataset, (B) the Markov et al (2012) macaque dataset, and (C) the meta-analytically collated macaque dataset (CoCoMac). Each vertical line represents one region pair, pairs are ordered by average weight of their connection. A thinner band indicates less variable values. The collated macaque dataset contains values in {1, 2, 3}; points have been jittered for visualisation purposes. (D-F) *V* = the variance of weights between repeated measures of the same connections divided by the overall variance of connection weights in the data.

The number of region pairs for which there were at least 2 non-zero weights was 492 for the multi-experiment mouse dataset; 153 for the multi-experiment macaque dataset; and 300 for the meta-analytically collated macaque dataset. The corresponding weights are shown in Fig 2. For the mouse dataset, variance for a region pair was *V* = 0.35 times as large as the total variance in the dataset. The multi-experiment macaque dataset was less variable (*V* = 0.13), whereas the collated macaque dataset was more variable, with more within-pair variance than between-pair variance (*V* = 1.3). This result remained unchanged when we restricted the region pairs for which the mean was calculated, or when not log-transforming the measured weights (S1 Appendix). Fig 2 further shows that in the mouse dataset, variance is particularly large for weaker connections: this might well be because measured signal is dominated by false positive noise. Computing the same measure as before with variance calculated over the 50% or 10% weakest weights leads to a stark increase: *V*_0.5_ = 0.54, *V*_0.1_ = 1.3. This effect was not there for the macaque: *V*_0.5_ = 0.16, *V*_0.1_ = 0.12.

### Estimation of mouse brain connectome

#### False positives due to automated segmentation

**Fig 3:**
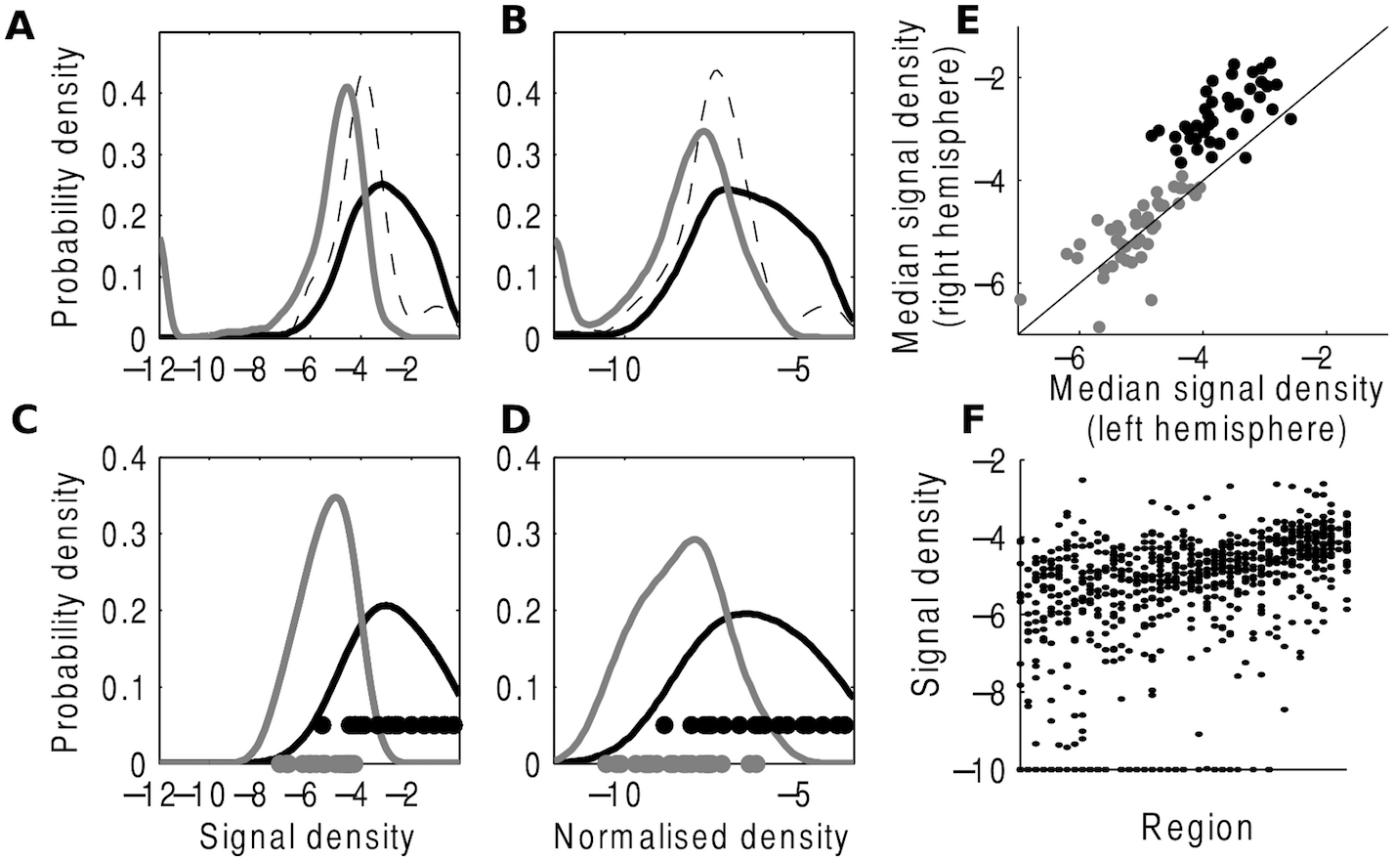
Characterisation of false positive distribution in the AIBS mouse dataset. (A-B) Distribution of all 43 × 2 × 20 algorithmically derived values for the 43 cortical areas in both hemispheres for 20 evaluated experiments, assigned as (black solid) true positive, (grey solid) false positive or (striped) non-evaluated. (A) The signal density *O*_*ej*_/*V*_*j*_ corresponds better to the manual assignments than (B) the normalized connection density *O*_*ej*_/*V*_*j*_/*I*_*e*_ (normalized Mann-Whitney U statistics: 0.88 and 0.81). (C-D) The same distribution for the 2 × 20 values from the primary visual area. Actual values are denoted by dots. (E) Comparison of median signal density for the 43 cortical regions and their contralateral homologue, for (black) true positive and (grey) false positive values. Data points for both assignments are strongly correlated across the two hemispheres (true positive: *r* = .60, *P* < 0.001, false positive: *r* = .73, *P* < 0.001). For true positives, right hemispheric values are significantly larger than left hemispheric (binomial test, *p* < .001). This is not the case for false positives (*P* > 0.1). (F) Density values for each of the 43 regions, combining the two hemispheres, ordered on median density. Values are distinct for different regions (Kruskal-Wallis: *P* < 0.001) but not for contralateral region pairs (uncorrected Mann-Whitney: *P* > 0.1 for all but one region).

We tested our predictions regarding the noise distribution, i.e. the signal found by the AIBS segmentation algorithm deemed to be false positive by expert curation. Firstly, we computed the normalized variables *U* for signal volume *O*_*ej*_, signal density $\frac{O_{ej}}{V_{j}}$ and normalized signal density $\frac{O_{ej}}{V_{j}I_{e}}$ as *U* = 0.86, *U* = 0.88, *U* = 0.81, respectively. This confirms that the signal density shows most overlap with expert curation of false positive signals (Fig 3A–3D). Secondly, a Kruskal Wallis test showed the distribution of noise-generated values differed significantly between regions (*P* < 0.001) (Fig 3E). Thirdly, we found no evidence of pairwise differences between contralateral homologues (uncorrected *P* = .03 for one region, *P* > .1 for all others, 43 comparisons performed). This was significantly different from the differences expected when comparing 43 random pairs (*P* < 0.001), where the minimum number of pairwise significant differences was 10 (over 1000 permutations). Furthermore, for both the true positive and false positive signal densities, the median signal densities for bilaterally symmetric regions were significantly correlated across the two hemispheres (*P* < 0.001; Fig 3F). However, for the true positive values, right hemispheric signal densities were significantly larger than left hemispheric signal densities (binomial test, *P* < 0.001). We would expect this difference for signal reflecting true connections as the injected hemisphere will show higher densities than the non-injected hemisphere. As expected, right hemispheric signal densities were not significantly larger for false positive signal densities (*P* > 0.1).

#### Connectivity estimates

**Fig 4:**
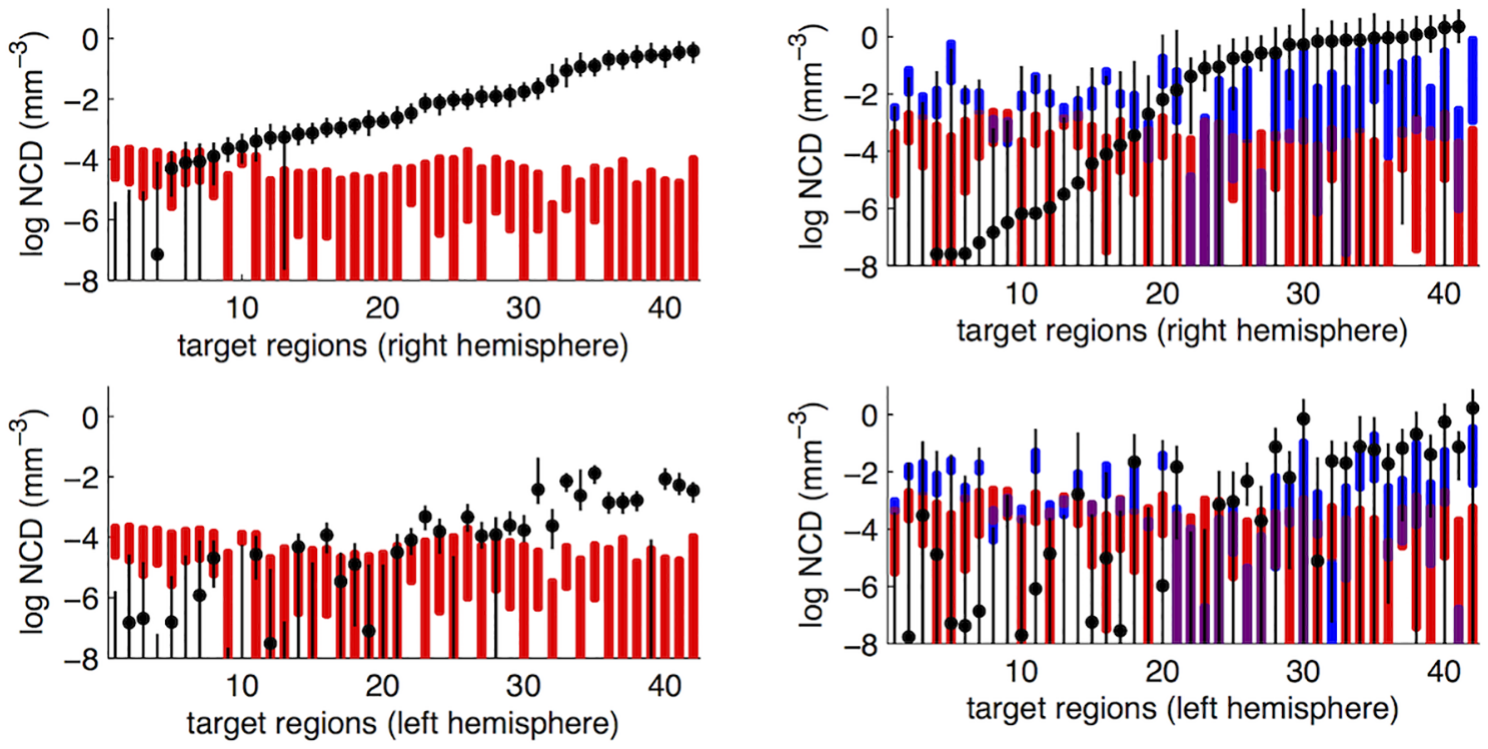
Representation of connection weights (normalized connection density, NCD) and thresholds for (left) primary visual area and (right) dorsal anterior cingulate area. Connections to (top) ipsilateral and (bottom) contralateral target regions, sorted by ipsilateral weights. (Black) Log connection weights and 95% credibility intervals (CI) are overlaid on the (red) noise threshold, due to low specificity of the automated segmentation algorithm, and the (blue) co-injection threshold, due to co-injection of several regions in one experiment. The noise threshold is identical for contralateral homologue target regions. A connection weight can only be assessed when it is stronger than the two thresholds, which is reflected in the large CIs below the thresholds. Note the absence of any estimate for the right-most connection in the top right panel; a connection cannot be assessed if the target region is co-injected with the source region for every experiment.

In the mouse dataset, 31 of the 43 cortical regions were injected with at least half the injection volume in at least one experiment. We obtained point estimates and credibility intervals for 2,627 out of 31 × 86 − 31 = 2,635 (99.7%) possible pair-wise connections; see S3 Table for full connectivity matrix. Self connections were not measured, the remaining 8 connections (*i*, *j*) could not be measured as the target region *j* received part of the injection for all experiments where *i* was injected. *σ* was estimated as 0.72 (95% credibility interval (CI): 0.69, 0.76). As a simple check of this estimate, we compared it to the standard deviation obtained for the raw normalized connection densities for the most often injected regions: visual primary region (32 injections), primary motor region (13 injections) and secondary motor region (8 injections). We found these values to be 0.69, 0.83 and 0.79, indicating that our estimate of *σ* is sensible. Fig 4 illustrates the estimates of *μ*_*ij*_, as well as thresholds found, for source regions *i* the primary visual area and the dorsal anterior cingulate area; see S1 Fig for other regions. The primary visual area has been injected 32 times with at least 50% of the injection volume. We found the CIs were small. However, it is impossible to estimate weights smaller than the noise thresholds. This is reflected in larger CIs for sub-threshold weights. The dorsal anterior cingulate area has been injected 3 times with at least 50% of the injection volume. However, all of these were co-injections with the dorsal anterior cortical region receiving less than 60% of total injection volume, and ventral anterior cingulate cortex receiving most of the remaining injection volume. This makes it difficult to estimate the connections of the two regions separately, which is reflected by high co-injection thresholds (Fig 4). For this reason, only the strongest connections of the dorsal anterior cingulate area can be precisely estimated, with many connections showing large credibility intervals.

### Cumulative network density estimation

**Fig 5:**
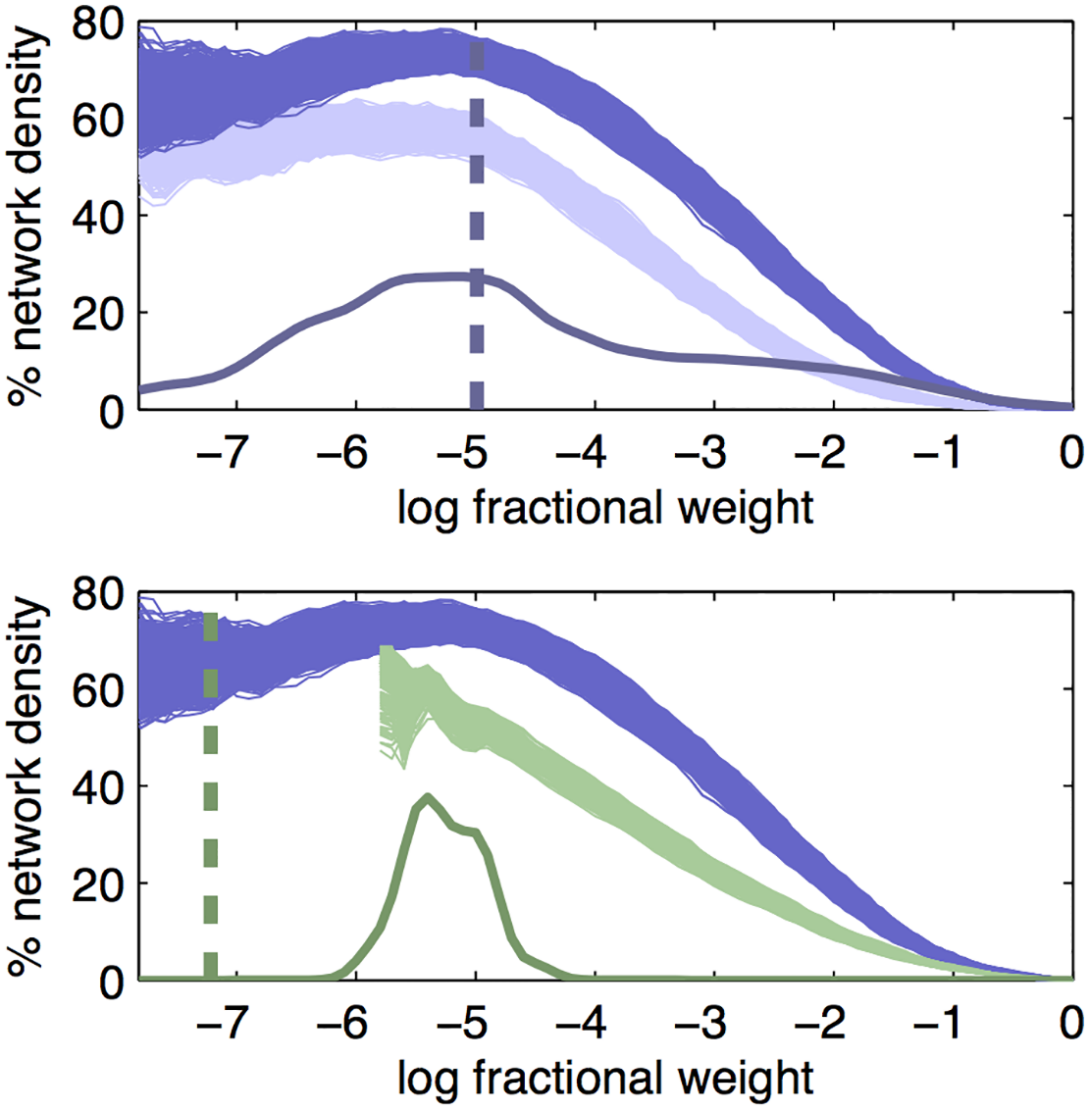
Network density of the cortical brain network for mouse and macaque. Shown is the percentage of fractional weights stronger than a threshold, as a function of the threshold used, separated in two panels for clarity, for (dark blue) mouse intrahemispheric, (light blue) mouse interhemispheric and (green) multi-experiment macaque intra-hemispheric connections. The thick solid lines give the density of thresholds for all connections. This distribution is wide for mouse (blue), which features both high-weight co-injection thresholds and low-weight region-specific noise thresholds. It is much narrower for macaque (green), as the threshold is 1/(number of neurons measured per experiment), which changes over only one order of magnitude between experiments. The dashed vertical lines give an estimated lower bound of the mean contribution of a single projecting neuron, for (blue) mouse and (green) macaque.

Fig 5 shows our estimates of overall intra- and inter-hemispheric network density *d*(*x*) in the AIBS dataset on the mouse and the Markov et al (2012) dataset on the macaque. To calculate *d*(*x*), we only consider those connections whose noise and co-injection threshold is lower than *x*. We see that overall the mouse has a higher network density than the macaque. We estimate the intrahemispheric network density as 73% (95% CI: 71%, 75%) for mouse and 59% (95% CI: 54%, 62%) for macaque. The interhemispheric network density for mouse was found to be 57% (95% CI: 54%, 59%). Note that our estimates are slightly lower than the sample mean weights [10] because we consider the log-transformed measurements, and *E*[*log*(*X*)] ≤ *log*(*E*[*X*]) (Jensen’s inequality). We find similar values (e.g. mouse intrahemispheric 71% (95% CI: 67%—75%)) when we adjust for the 12 uninjected cortical areas (S1 Appendix).

### Biological interpretation

From Fig 5 it can be seen that the mouse network density increases slowly as the threshold *x* is lowered from 0 to -1, sharply and nearly linearly as *x* decreases from -1 to around -5, and then reaches a plateau. The threshold at which the plateau is reached is close to the estimated lower bound of the fraction of signal due to a single projecting neuron (Fig 5). The apparent decrease in network density around *x* = −7 is somewhat surprising, as the true network density is a decreasing function of *x*. However, our estimate of *d*(*x*) is based on only those connections that can be measured at this level, i.e. whose median noise threshold and median co-injection threshold are smaller than *x*. Thus, the estimate of *d*(*x*) is based on a decreasing number of connections as *x* decreases. The remaining connections at *x* = −7 are to the few target regions that have median noise threshold smaller than -7, which can bias the estimate of *d*(*x*). The macaque network density shows a similar first slow and then rapid linear increase, but does not reach a plateau. None of the macaque experiments has enough sensitivity to reach the estimated lower bound of the fraction of signal due to a single projecting neuron (Fig 5), possibly reflecting the small injection volume relative to the volume of the injected regions [10].

### Topological analysis of weak connections

**Fig 6:**
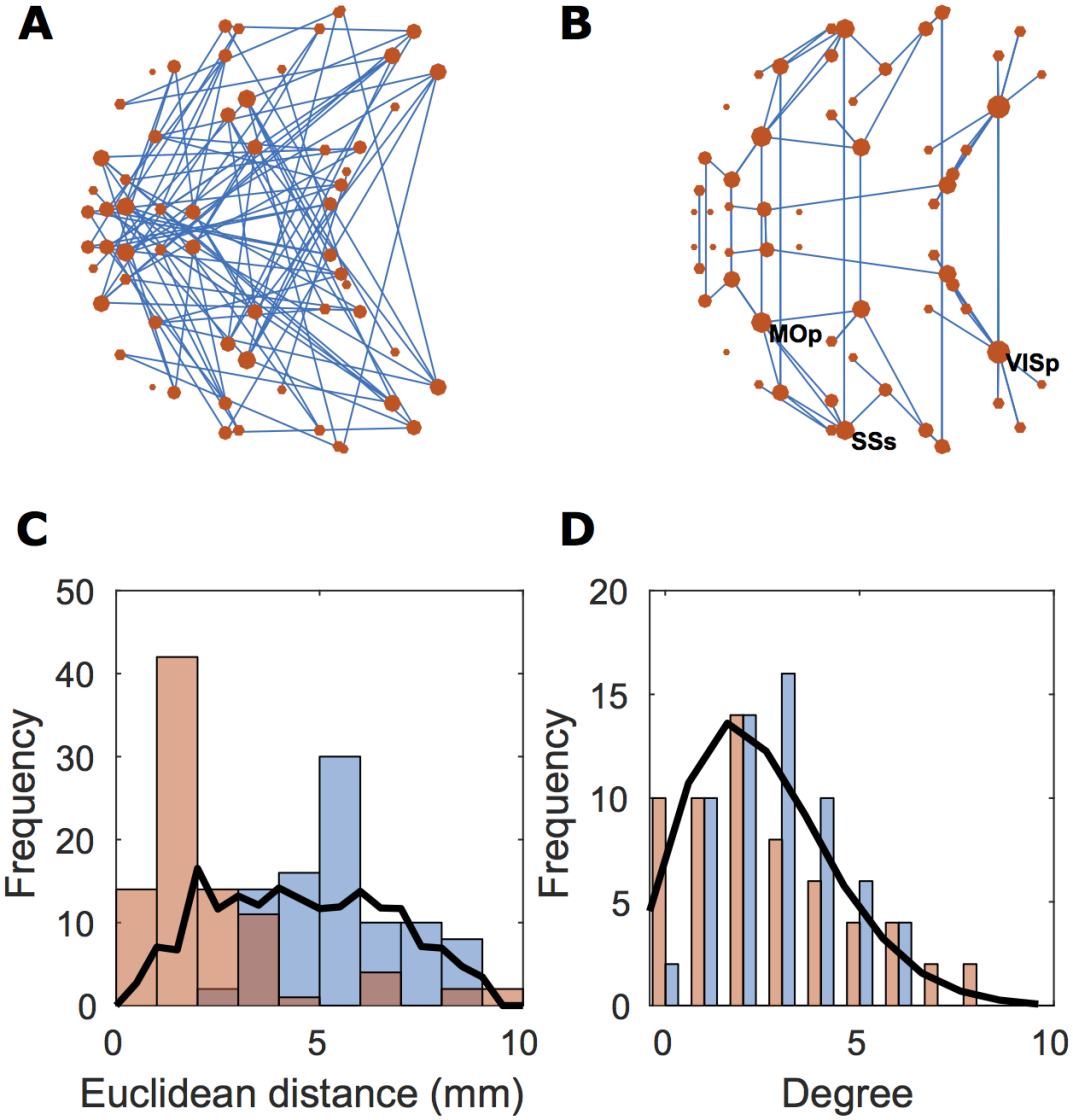
Properties of the 5% weakest and 5% strongest edges of the mouse cortical network. (A,B) Axial view of the mouse cortical networks, red dots represent brain regions, blue lines represent the connections between them. Drawn are the (A) 5% weakest or (B) 5% strongest edges. Dot size corresponds to degree, the total number of incoming and outgoing edges connected to a node. In (B), the three nodes with highest degree have been labeled: VISp, primary visual area; MOp, primary motor area; SSs, supplemental somatosensory area. The strong connections are spatially organized, mainly connecting spatially adjacent or contralaterally homologous regions. The weak connections span longer distances and are topologically more random than the strongest connections. (C) The distance distributions for (blue) the 5% weakest edges, (red) the 5% strongest edges, and (black) a random graph of the same size and connection density. (D) The degree distributions for the weakest and strongest connections of the mouse connectome, and a comparable random graph, color-coded as in (C).

The 5% strongest (mean log weight (*n*_*ij*_)∼10^−0^ mm^-3^) and the 5% weakest connections (mean log weight (*n*_*ij*_)∼10^−5^ mm^-3^) were mapped separately in anatomical space (Fig 6). It is clear by inspection that the strongest connections were more locally and topologically clustered whereas the weakest connections were more random topologically and subtended longer connection distances spatially. The distance distribution is shifted to the right for the weakest connections and the degree distribution has a fatter tail, implying greater probability of high degree hubs, for the strongest connections.

**Fig 7:**
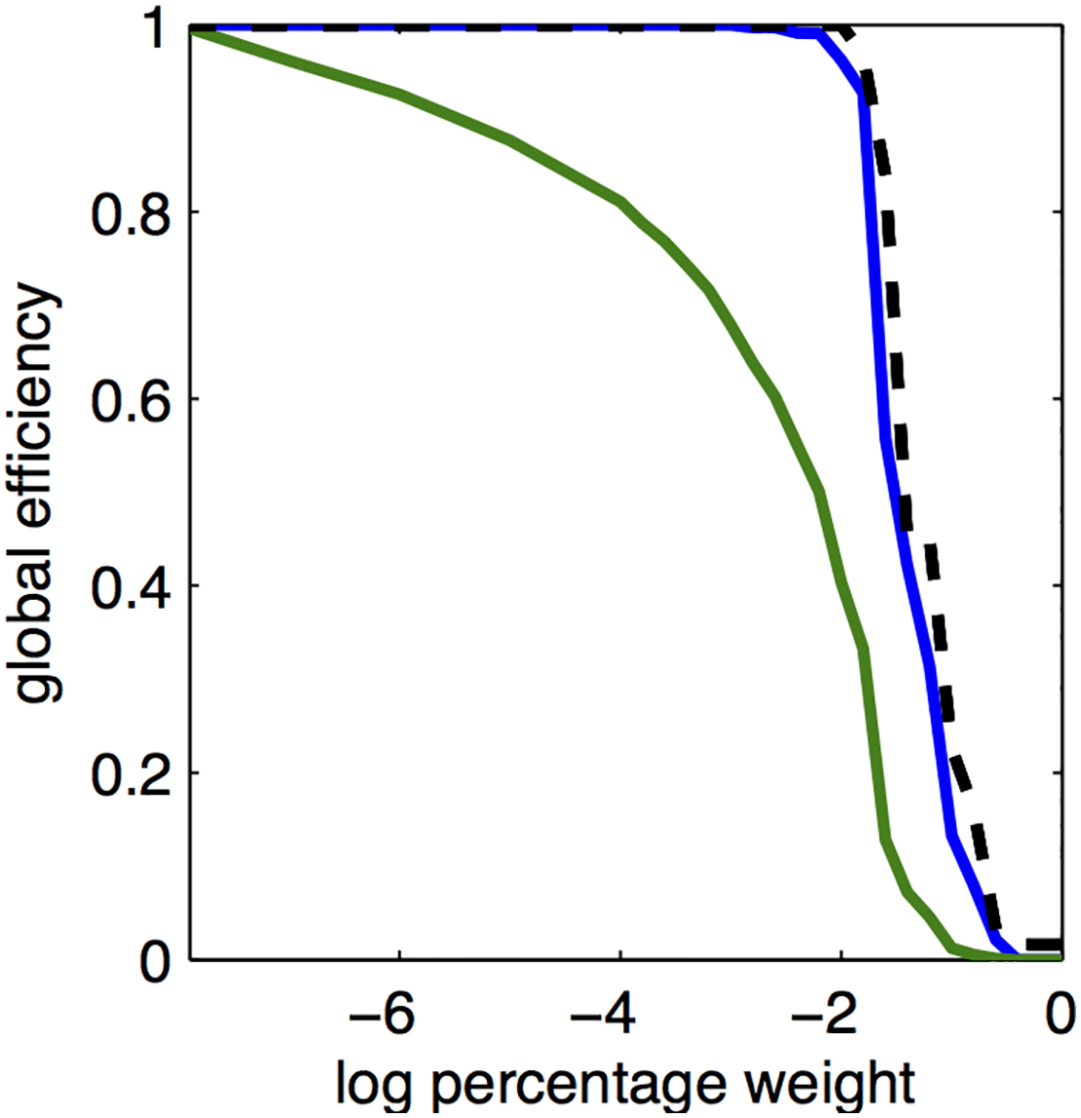
Global efficiency of the mouse connectome modelled by weighted and binary graphs. The global efficiency of the thresholded mouse network increases as the weight threshold is reduced, for (blue) the weighted graph and (green) the binarized graph. Dashed line gives the fraction of nodes contained in the largest connected component. In the weighted graph (blue), the weaker weights have little impact on the global efficiency or connectedness of the network; whereas in the binary graph (green) the weakest edges incrementally increase topological integration of the network.

Considering the topology of the whole connectome, we used weighted and binary graph models to investigate how global efficiency and the fractional size of the largest connected component (two metrics of network integration) behaved as a function of variable threshold for edge identification. For both analyses, the network becomes fully connected, i.e. the fractional size of the largest connected component becomes 1, at high thresholds (*x* ∼ 10^−3^; Fig 7). In the weighted graph analysis, the network reaches maximal efficiency similarly quickly, when only a relatively small proportion of strongly weighted edges have been included in the network. Addition of weak edges, by lowering the threshold, does not materially affect this metric of weighted graph topology. In the binary graph analysis, progressive lowering of the threshold is associated with a more gradual increase in efficiency, with incremental increases even at the lowest thresholds inclusive of the weakest connections.

## Discussion

We have assessed the variability of three large, quantitative tract-tracing datasets on the axonal connectivity of the mammalian cortex. We found lower variability for expertly curated data on the macaque than for algorithmically segmented data on the mouse; and highest variability for the meta-analytically collated dataset on the macaque. We then articulated a statistical framework to estimate the connection weights, and quantify the uncertainty in these estimates. We applied this approach principally to the the multi-experiment mouse dataset provided by the Allen Institute for Brain Science (AIBS) [9] and estimated the mouse connectome (S3 Table), accounting for false positive signals generated by algorithmic segmentation and for co-injection of tracer into more than one source region in a single experiment. Finally, we have explored some of the biological implications of these results. We found that the connection density of the mouse cortex was considerably higher than previously reported (73%) and that the weakest connections, probably representing no more than a few axonal projections, have a relatively random organisation with modest impact on the topology of the mouse cortical connectome.

Variability in repeated measurements of the same connection arises as a combination of inter-animal biological variability and measurement errors. We quantified the variability of each dataset as the mean variance of repeated measurements of the weight of the same connection normalized by the variance of all connection weights. Ideally, the former should be small compared to the latter. The multi-experiment macaque data from Markov et al [10] are clearly much less variable than the multi-experiment mouse data from Oh et al [9], with variability values of 0.13 and 0.35 respectively. This finding can be partly explained by the inhomogeneity of brain regions. Markov et al. performed repeat injections in the exact same position in the brain region specifically to assess variability, whereas Oh et al. performed injections in variable positions within each source region, specifically in order to increase coverage of cortex by tracer injections. Worryingly, the CoCoMac dataset has variability of 1.3, indicating that there is greater variance of repeated measurements than of all connection weights. This high value may be attributable to technical limitations in the algorithm employed to map all primary studies to the same anatomical atlas [23]. More fundamentally, it may reflect the inevitably lower reliability of measurements made in different labs using different experimental procedures (such as differing planes of section) to measure nominally identical connections [29]. In any case, it seems clear that the pioneering value of meta-analytic approaches to mammalian cortical connectomics has been surpassed by the much higher reliability of next-generation tract-tracing datasets compiled from multiple experiments conducted according to standard experimental protocols.

Our analysis has made clear how the mouse data provided by Oh et al. [9] suffers from at least two sources of noise. Firstly, the segmentation algorithm employed generates many false positive signals. We have quantified the distribution of this segmentation noise, constructing a per-region threshold for the minimum connectivity weight that is sufficient to refute the null hypothesis. Any connection to this region that would result in signal weaker than the threshold cannot be distinguished from positive-mean segmentation noise. The second source of noise in the AIBS datasets arises from co-injections. Among the large number of injection experiments provided by the AIBS—here, 489 experiments each involving a single localised cortical region of anterograde tracer—not all the injections have been constrained to a single anatomical region as pre-defined by an atlas or template. In some experiments, a third or more of total tracer volume will have been injected into one or more spatially adjacent cortical areas. Co-injection of two regions, for example, makes it difficult to disentangle the specific connectivity of each of these two source regions to the same target region. Statistically, this uncertainty about the weight of pair-wise axonal connectivity estimated for co-injected source regions can be represented by a co-injection threshold, which is unique for each pair of regions, and depends on the co-injection pattern of the experiments. Our statistical framework takes into account these two thresholds, and correctly reports high uncertainty when connections are weak relative to the two thresholds.

We have found the intra-hemispheric network density, i.e. the fraction of possible pair-wise connections that exists, of the mouse to be 73% (95% CI: 71%, 75%). This estimate appeared robust; adjusting for uninjected regions using a linear model that accounted for region size and neuronal density yielded a very similar density of 71% (95% CI: 67%—75%; S1 Appendix). These estimates of cortical connection density are higher than previous estimates in mouse [34%-41% (isocortex) [8]; 35.4%-53.5% [9]], but closer to recent estimates of cortical intra-hemispheric connection density in macaque [62% [10]]. There are two reasons we would a priori expect to find a somewhat higher network density for the mouse than macaque. Firstly, the cost of long-range connections increases at a higher rate for larger brains [21], thus we would expect higher network density for smaller brains. Secondly, the mouse has fewer identified cortical areas, and network density increases with decreasing number of brain areas. Thus, the finding of 73% network density seems to corroborate claims that the mammalian cortical network density has long been under-estimated [17]. As a technical caveat, we should note that inter-areal connections in mouse can be completely embedded in grey matter and that the anterograde tracer employed is sensitive to these axonal projections passing through a cortical area en route to termination in another area, which could potentially lead to an overestimation of the network density in mouse. Nevertheless, it is clear that advances in tract-tracing technology have enabled detection of axonal connectivity over 6 orders of magnitude with just-detectable connections constituting very weak links compared to the nearly million-fold greater connectivity weight of the most strongly weighted connections.

The biological meaning of such unprecedentedly weak axonal connectivity is not immediately clear. We computed a simple approximation of the connectivity weight attributable to a single axonal projection, by dividing the total number of neurons in mouse cortex (4 × 10^6^) by the number of cortical regions (43) so that average total tracer signal from any region was attributable to at most $\frac{4\times 10^{6}}{43}$ axonal projections. This single axon threshold corresponds to connection weights ∼10^−5^. The single axon threshold corresponds reasonably well with the point at which the estimated mouse network density, both intra- and inter-hemispheric, reaches a plateau. This observation is consistent with the log-normal distribution of connection weights [16, 30] extending to the scale of a single axonal projection, which would maximize the dynamic range of connection weights hypothesised to be important for brain function [31].

Weak axonal projections, down to the limit of a single axon connecting two cortical areas, should not be discounted as organisationally trivial. In social and other complex networks [32] weak links are well-recognised to serve important integrative functions in social functions, e.g., mediating information transfer, or gossip, between otherwise isolated cliques. In the macaque brain, it has been consistently argued that weak connections may have functionally important effects on brain dynamics by orchestrating oscillatory coherence of anatomically distributed cortical areas [10, 14, 16, 17, 33]. In the mouse connectome, the weakest links were topologically more random than the strongest links, and they traversed greater anatomical distances than the more locally clustered or lattice-like strongest links. These properties are conceptually consistent with the weakest links of the mouse connectome playing a similarly integrative role to weak links in social networks [34]. However, it is difficult to say how functionally important a single axonal projection to a cortical area comprising ∼10^5^ neurons is likely to be in real-life. In a graph theoretical analysis weighted by axonal connectivity over 6 orders of magnitude, the topology of the network is dominated by strongly connected edges. The network is node-connected and has maximal global efficiency at a 10^−2^ weight threshold, equivalent to a network density of 7.5%. Further relaxation of the weight threshold does not substantially change the global topological properties established by the most strongly connected edges, consistent with results found in macaque [14]. However, if the network is modelled as a binary graph, effectively equalizing all true connection weights, then we can see theoretically expected incremental increases of network efficiency and integration as progressively weaker edges are added by relaxation of the edge threshold down to the minimum imposed by experimental noise. Future studies may explore this question in more depth by explicitly considering the topological properties of those weak connections that are estimated to exist with high confidence.

One way of thinking about these observations is that the weakest links add randomness to mouse connectome topology and integrative capacity to mouse connectome function. Prior results on retrograde fluorescent tract-tracing data on the macaque [10, 14, 16, 33] likewise found that the weaker connections (new found projections) were topologically integrative; but also demonstrated that the new found projections shared an anatomically specific profile of inter-areal connectivity in common with stronger (previously known) connections between cortical areas. The anatomical specificity of new found projections in the macaque cortex suggests that they are not as randomly organised as the weakest connections of the mouse cortex reported here. However, it is important to recognise that new found projections constituted approximately the weakest 36% of all inter-areal connections (on average over areas) in the macaque [33]; whereas we have focused on the weakest 5% of all connections in the mouse. It is plausible that the topological randomness of weak links may increase monotonically as a function of increasing weakness. In other words, the greater randomness of these results on the mouse may not be attributable to inter-species differences but rather to our focus on a smaller subset of more extremely weak links than the relatively larger subset of less weak new found projections. Future comparative studies would be useful to test this prediction more rigorously. Future studies of the mouse connectome might also explore the generative hypothesis that the random topology of the weakest links reflects their formation by stochastic processes of cortical network development. For example, synaptic connectivity of cortical neurons peaks in early post-natal years for mammals and there typically follows a prolonged phase of synaptic pruning or deletion of functionally unimportant, aberrant or over-exuberant synaptic connections and associated axonal projections. Therefore, one possible developmental mechanism for the randomly organised weak links of the adult mouse connectome could be that they reflect what remains after pruning of functionally prioritised connections. A testable prediction of this hypothesis is that the weight of the weakest adult connections should rise and then fall during post-natal connectome development.

In conclusion, we have provided a statistical framework to analyse tract tracing data, yielding estimates of connection weights and their associated uncertainty. We have provided these estimates and thresholds for the mouse connectome, such that they may be analysed by other researchers (S3 Table). We have drawn attention to the higher-than-expected maximum cortical connection density of the mouse, attributable to the precision of the measurements allowing resolution of tracer signals in the order of a single axon. These very weak inter-areal axonal projections are not yet completely understood biologically. Like weak links in social networks, they could be topologically integrative and important for global information transfer and/or the weakest (ultimately single axon) projections could be the lucky survivors of a developmental pruning process programmed to entirely eliminate them. It seems likely that functional importance will go with increasing axonal connectivity weight, so the very weakest connections are least likely to be functionally important for inter-areal communication; but the minimum weight needed for functional importance, or the functional threshold on axonal connectivity, is not yet known.

## Supporting Information

S1 Table List of the 43 Allen Reference Atlas mouse cortical regions used. (CSV)

S2 Table Manual assessment of algorithmically segmented data from 20 tract-tracing experiments, kindly provided by the Allen Institute for Brain Sciences. These data correspond to figure S7 from [9].
(XLSX)

S3 TablePoint estimates and credibility intervals for the mouse cortical network.Provided are the estimated connection weights ($\bar{\mu }$), as well as the estimates of the co-injection threshold and noise threshold.(XLS)

S1 Fig Visualization of connection weight and threshold estimations for all regions not in Fig 4. (PDF)

S1 Appendix Sensitivity analyses on 1) computation of connectivity weight variability *V*, 2) use of informative priors and 3) correction for uninjected regions in density estimation. (PDF)
