# Supplementary figures and images for: Statistical Analysis of Tract-Tracing Experiments Demonstrates a Dense, Complex Cortical Network in the Mouse
Source: PLoS Comput Biol. 2016 Sep 12;12(9):e1005104. doi: 10.1371/journal.pcbi.1005104 (PMC5019374; doi:10.1371/journal.pcbi.1005104)

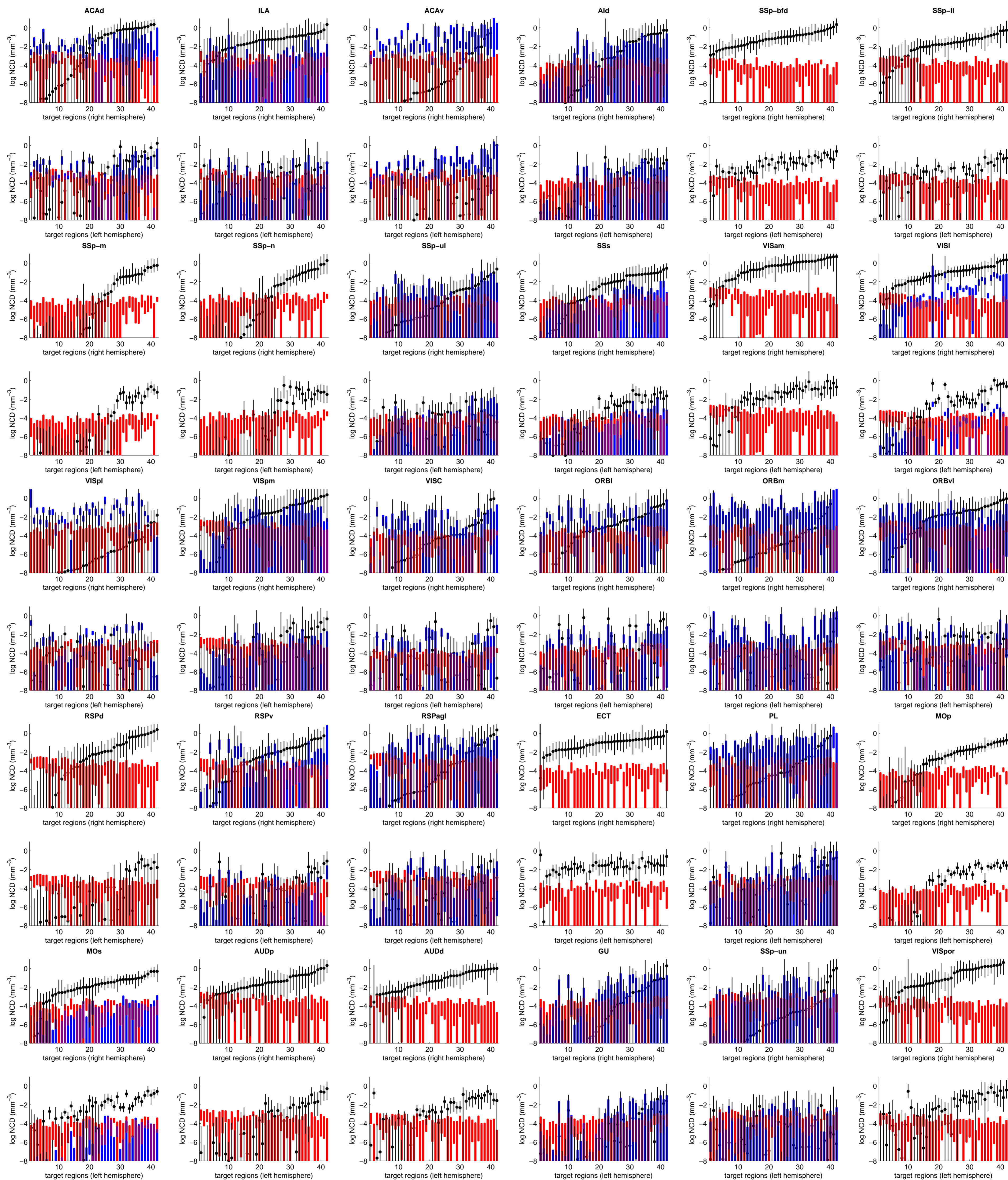

Supplement: S1 Fig — (PDF) [file pcbi.1005104.s004.pdf]
