# Supplementary material for: Statistical Analysis of Tract-Tracing Experiments Demonstrates a Dense, Complex Cortical Network in the Mouse
Source: PLoS Comput Biol. 2016 Sep 12;12(9):e1005104. doi: 10.1371/journal.pcbi.1005104 (PMC5019374; doi:10.1371/journal.pcbi.1005104)
Supplement: S1 Appendix — (PDF) [file pcbi.1005104.s005.pdf]

Appendix

Statistical analysis of tract-tracing experiments demonstrates a dense, complex cortical network in the mouse

Rolf JF Ypma, Edward T Bullmore

Sensitivity analyses

Robustness of variability of axonal connectivity weights measure V

In the main text, we compute the variability of axonal connectivity weights for three different datasets as $V = \frac{E(\overline{v})}{\mathrm{Var}(\overline{m})}$, where v is the variance of connections measured at least twice, and m is the mean of all connections measured. Different definitions of variability exist, each measuring a slightly different aspect of the same concept. Here, we examine the robustness of our results with respect to two of these, both addressing addressing a potential shortcoming of the definition we employed.

Firstly, a bias could have been introduced by the current definition as v and m are not computed from the exact same set of connections, e.g. if the connections measured only once would be more or less variable. We therefore compute V_2, which is identical to V except that we compute the means m_2 over connections measured at least twice, such that $v_2 = v$ and m_2 are computed over the same set of connections. Secondly, the current definition ignores information by discarding measurements of 0, which is necessary due to our log-transform of weights. We therefore compute V_3, which is identical to V, except that all weights x are first transformed as 10^x. In other words, in computing V_3 the weights from the two multi-experiment datasets are not log-transformed (as they were to compute V), and the weights from the macaque dataset are transformed as follows: $0 \to 1, 1 \to 10, 2 \to 100, 3 \to 1000$. We then compute the means m_3 and variances v_3 as before for all pairs of regions whose connection was measured at least twice, for which at least one measurement was non-zero (i.e. the connection exists). We include weight 0 in these computations. We then compute V_3 as the log transform of the mean variance divided by the variance of the means

$$V_3 = \log_{10}\left(\frac{E(\overline{v_3})}{\mathrm{Var}(\overline{m_3})}\right)$$

Results In the main text, we found the mouse dataset's consistency ($V = 0.35$) to be intermediate between that of the multi-experiment macaque ($V = 0.13$) and collated macaque ($V = 1.3$). Computing the means over all connections measured at least twice resulted in very similar results: $V_2 = 0.33$ (mouse), $V_2 = 0.14$ (macaque), $V_2 = 2.0$ (collated macaque). The large increase in the collated macaque data is due to its integer nature and high inconsistency; repeatedly measuring a connection is likely to lead to a less extreme mean value for the connection. Thus, excluding connections measured only once will lead to a lower variance of the means. Likewise, our second approach of including zero measurements by taking the log transform later, leads to similar results: $V_3 = 0.20$ (mouse), $V_3 = 0.13$ (macaque), $V_3 = 1.7$ (collated macaque).

Use of informative priors in estimation

In the main text, we used a Bayesian approach to optimally estimate connection weights and associated uncertainty from viral tracing data. We used uninformative priors (uniform distributions on our parameters $\overline{\mu}$ and σ). Such an uninformative prior assures that the posterior distribution optimally reflects the information present in the data. One advantage of the Bayesian approach is that it can easily incorporate information from other sources, such as an independent set of tract tracing experiments, by adjusting the prior distributions. To test the impact of using informative priors, we repeated our analysis, assuming prior knowledge for the weight of all connections. In particular, we used a normal $Norm(-2,2)$ distribution for all μ_{ij}. This prior is relatively close to the full posterior distribution of weights from our full analysis, and expresses the belief that all weights should be similar to this distribution. We would expect that weights for which much information is present in the data would show a posterior distribution close to the the posterior found in the initial analysis, i.e. would not be affected much by the prior. On the other hand, we expect weights for which little information exists in the data to have a posterior distribution close to their prior distribution.

Results Fig. S_A1 shows the weights found for the primary visual and dorsal anterior cingulate area, in the exact same format as Fig. 4 in the main text. As expected, we see that weights well characterised by the data, i.e. strong weights with small credibility intervals, have a similar posterior distribution when using uninformative priors (Fig. 4) or informative priors (Fig S_A1). In contrast, the weights for which little information is present, i.e. the weak weights that that fall below their associated thresholds and have large credibility intervals, show a posterior distribution much closer to the prior distribution. In particular, many weights that were previously estimated to be smaller than -4 mm^{-3} are now estimated at the higher range of their noise and coinjection threshold, at around -3 mm^{-3}. This effect can be seen even more clearly in Fig. S_A2, where we plot the complete distribution of weights (μ_{ij}) and the priors used. When using an informative prior, it is mainly the weaker weights that become larger, with a posterior distribution closer to the prior distribution. Strong weights remain at the same values as when using uninformative priors.

These results illustrate that 1) our overall results are relatively robust to inclusion of more informative priors and 2) informative priors can be sensibly used in estimating the connection weights, paving the way for future studies combining different datasources.

Correction for uninjected regions

In our analysis, we only obtain estimates for efferent connections of the mouse connectome from 31 of the 43 cortical brain regions, as only these were injected with at least 50% of the injection volume for at least one experiment. Thus also our estimate of the density of the full connectome ignores the connections originating from these remaining 12 regions. This could bias our results, as these regions may have different anatomical properties affecting their connectedness, e.g. they are smaller on average than the injected regions (Table S1). As larger regions tend to be more well connected, our estimates may be overestimates of the true density. Here, we aim to correct for this possible bias by estimating the connection density of the 12 uninjected regions using a set of anatomical descriptors. Although this is a crude way of estimation, it allows us to assess the magnitude of this possible bias. We employ a linear model that includes three available anatomical descriptors: region size, neuronal surface density and neuronal density.

We compute the adjusted network density function $d_a(x)$, defined as the percentage of all possible connections that are of at least weight x, corrected for the uninjected

regions. First, for any x, we calculate this density for $C(x)$, defined in the main text as the subset of the 31 injected regions that have at least one connection for which the thresholds are smaller than x. We then fit a multiple regression model on these densities, with region size, number of neurons per surface area and number of neurons per unit of volume as explanatory variables. The latter two were obtained from Herculano-Houzel et al. [1]. These authors provide these measures for coarse areas of the mouse brain. We mapped our 43 regions to their subdivision and assigned the regions the corresponding values. As our regions were subsets of their subdivisions, several of our regions are assigned equal values (Table S1). From the regression model we obtain estimates of the densities of the 12 uninjected regions. Let $d_u(x)$ be the average of these estimates. We then set our adjusted network density as the weighted average over all estimated densities

$$d_a(x) = \frac{31}{43}d(x) + \frac{12}{43}d_u(x)$$

Results Fig. S_A3 shows results found, in the same format as Fig. 5 in the main text. We find that adjusting for uninjected regions leads to somewhat lower and substantially more uncertain estimates: intrahemispheric connection density 71% (95% credibility interval (CI): 67% - 75%) (main text: 73% (95% CI: 71%, 75%)), interhemispheric 52% (95% CI: 48%, 56%) (main text: 57% (95% CI: 54%, 59%)). Surprisingly, although the adjusted density $d_a(x)$ is smaller than $d(x)$ for most x, it becomes larger as x becomes smaller than around -6. This may be due to a bias caused by the small number of regions that have a noise threshold low enough that connections to them can be measured for these low x values. As these low-threshold regions are mainly visual regions, all mainly connected to other visual regions sharing a distinct set of anatomical properties such as high neuronal density, the linear model might gives biased estimates for the connectivity of the uninjected regions.

Although the simplicity of the model we here employed renders it difficult to make definitive statements on the connection density of the full cortex, the analysis strongly suggests that our conclusions on the high value of the connection density are unlikely to be affected by the presence of uninjected regions.

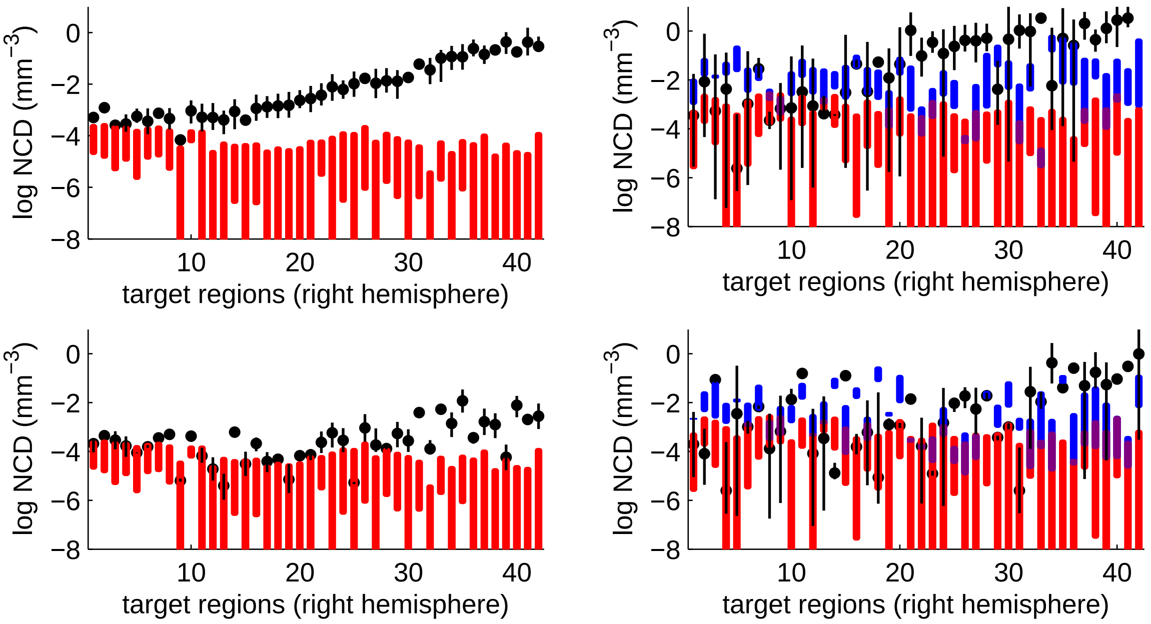

Figure S$_A$1. Representation of connection weights (normalized connection density, NCD) and thresholds for (left) primary visual area and (right) dorsal anterior cingulate area, when assuming an informative prior. This figure corresponds to Fig. 4 in the main text. Connections to (top) ipsilateral and (bottom) contralateral target regions are shown, sorted by the ipsilateral weights as originally found (Fig. 4, main text) to facilitate comparison. (Black) Log connection weights and 95% credibility intervals (CI) are overlaid on the (red) noise threshold, due to low specificity of the automated segmentation algorithm, and the (blue) co-injection threshold, due to co-injection of several regions in one experiment. The noise threshold is identical for contralateral homologue target regions, and remains unchanged when assuming an informative priors. Notice that few connections are estimated as being lower than their threshold: in this case the data will have limited information on the actual weight, and the posterior distribution will be similar to the prior distribution. Thus, the main difference with the results found when using an uninformative prior (Fig. 4, main text) lies in the posterior distribution of these weak weights.

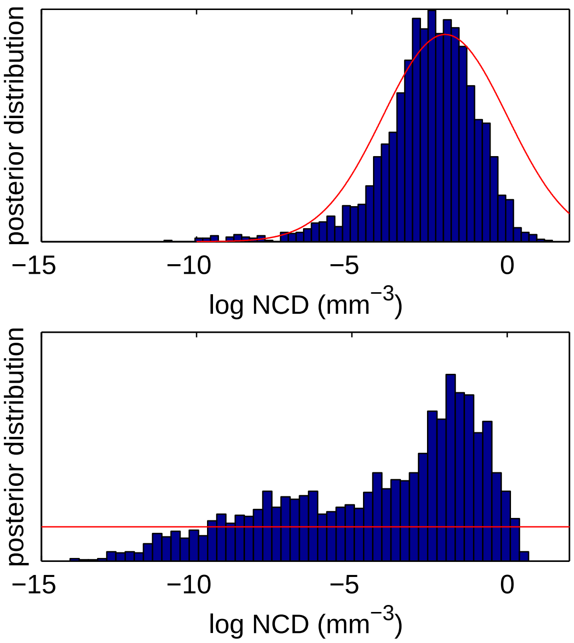

Figure S_A2. Distribution of connection weights and assumed prior distribution. (Blue) Histogram of connection weights and (red) the prior distribution assumed, for (top) informative prior and (bottom) uninformative prior used. We see the main effect of the informative prior is to increase the estimates of the weak, sub-threshold weights, for which little information is present in the data.

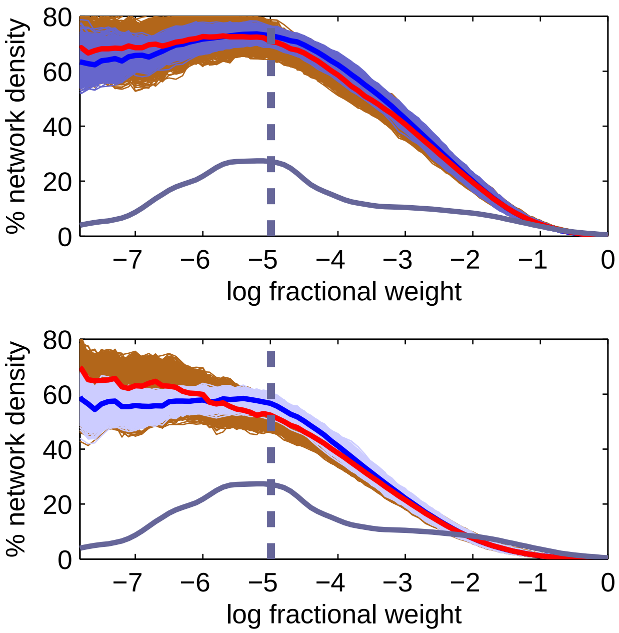

Figure S_A3. Network density of the cortical brain network for mouse (cf Fig 5 in main text). Shown is the percentage of fractional weights stronger than a threshold $d(x)$, as a function of the threshold x used, separated in two panels for clarity, for (dark blue, top) mouse intrahemispheric, (light blue, bottom) mouse interhemispheric and (orange) the same density adjusted for the possible bias caused by uninjected regions $d_a(x)$. The red and blue lines give the point estimates for $d(x)$ and $d_a(x)$, i.e. the median of the values from the MCMC sampled. The thick solid lines give the density of thresholds for all connections, the dashed vertical line gives an estimated lower bound of the mean contribution of a single projecting neuron. Adjusting for uninjected regions leads to lower estimates and more uncertainty, i.e. a wider distribution. The higher values for $d_a(x)$ for $x < -6$ are probably due to a bias caused by only a limited number of regions having noise thresholds < -6.

References

1. Herculano-Houzel S, Watson C, Paxinos G. Distribution of neurons in functional areas of the mouse cerebral cortex reveals quantitatively different cortical zones. Front Neuroanat. 2013 21 Oct;7:35.
